# Short Implants Versus Sinus Floor Elevation With Standard Implants in the Atrophic Posterior Maxilla: A Systematic Review and Meta-Analysis

**DOI:** 10.7759/cureus.116807

**Published:** 2026-09-23

**Authors:** Salma Abdallaoui Maane, Cesar Muelas Sanchez, Manuel Lanciego, Ana Marín Aguado

**Affiliations:** 1 Periodontics, Implantology, and Oral Surgery, Universidad San Pablo CEU, Madrid, ESP

**Keywords:** atrophic posterior maxilla, implant survival rate, marginal bone loss, maxillary sinus augmentation, meta-analysis, short dental implants, sinus floor elevation, systematic review

## Abstract

Posterior maxillary atrophy is a significant challenge in implant dentistry because of limited subantral bone height and proximity to the maxillary sinus. The two main therapeutic options are sinus floor elevation with standard implants (>8 mm) and short implants (≤6 mm). This systematic review and meta-analysis compared both techniques in terms of implant survival, marginal bone loss, and complication rates, following Preferred Reporting Items for Systematic Reviews and Meta-Analyses (PRISMA) 2020 guidelines. Electronic searches were conducted in PubMed/MEDLINE, Scopus, Web of Science, and Cochrane Central, including randomized controlled trials with at least one-year follow-up, published between 2014 and 2025. Eligibility was deliberately restricted to trials in which the control arm involved sinus floor elevation, excluding studies that compared short implants with longer implants placed in native bone of adequate height or in bone augmented by other techniques. Four publications corresponding to three independent cohorts were included (174 randomized patients); a fifth report of one of these cohorts could not be retrieved and contributes no data. All data were re-extracted in duplicate directly from the source publications. Survival was analyzed at the patient level; one cohort recorded no implant failures in either arm and therefore contributes no information to a risk ratio (RR), leaving 154 patients (83 short implants/71 standard implants) in the primary pooled analysis. Cumulative patient-level survival across the three cohorts was 97.8% for short implants and 98.8% for standard implants. Meta-analysis yielded a pooled RR of 1.00 (95% CI: 0.56-1.76, Hartung-Knapp adjustment; unadjusted interval 0.91-1.09, p = 0.92). On the failure scale, three failures occurred in total, 2/93 with short implants and 1/81 with standard implants, giving a Peto odds ratio of 1.70 (95% CI: 0.17-16.86). Substantial between-study heterogeneity was present (I² = 59%), with the two pooled cohorts pointing in opposite directions: 100% versus 95.0% in one and 96.0% versus 100% in the other. Marginal bone loss favored short implants in one cohort by a margin below clinical relevance and did not differ between groups in the other; the data were not pooled because radiographic methods and reporting formats differed across trials. Biological complications were consistently less frequent with short implants, with every failure recorded in a standard-implant arm attributable to graft-related complications.

The review cannot determine whether the two techniques differ in implant survival. With three failures among 174 randomized patients and two contributing trials, the confidence interval is compatible with a substantial advantage to either approach. The most consistent finding concerns morbidity rather than survival.

## Introduction and background

Oral implantology has transformed treatment for edentulous and partially edentulous patients, offering a durable, functional solution for restoring masticatory anatomy and physiology. Implant success nevertheless depends on the quantity and quality of bone available at the recipient site. Alveolar bone resorption following tooth loss, prolonged edentulism, or periodontal disease creates considerable challenges, particularly in the posterior maxilla [1,2].

Atrophy of the posterior maxilla is common among edentulous and partially edentulous patients, with prevalence increasing markedly with age, and its etiology is multifactorial. Tooth loss is the principal contributing factor, whether secondary to caries, periodontal disease, trauma, or planned extraction, and the duration of edentulism largely determines the extent of resorption. Smoking, diabetes, osteoporosis, chronic periodontal disease, and advanced age are recognized risk factors for severe atrophy. The Cawood and Howell classification, which distinguishes six stages of resorption, remains a reference tool for diagnosis [3,4].

The anatomical configuration of this region compounds the difficulty. The maxillary sinus is a pneumatized cavity within the maxilla, lined by the Schneiderian membrane. After tooth loss, secondary pneumatization may expand the sinus cavity at the expense of residual alveolar bone. The resulting subantral bone height is often insufficient for conventional-length implants, which generally require at least 8 to 10 mm of residual bone without augmentation. Preoperative cone-beam computed tomography (CBCT) is essential for quantifying residual bone height and planning treatment, and this anatomical limitation directly influences treatment choice [5,6].

Sinus floor elevation, first described by Boyne and James in 1980 [5], addresses this limitation by elevating the sinus mucosa, with or without graft material, thereby permitting placement of standard-length implants (>8 mm). Two approaches are described: the lateral window technique and the less invasive transcrestal approach introduced by Summers in 1994 [7]. The procedure is reproducible and predictable, with survival rates exceeding 90%, but it carries recognized drawbacks: an additional surgical step, increased postoperative morbidity, prolonged treatment time, and higher costs. Perforation of the Schneiderian membrane is the most frequent intraoperative complication, with a weighted incidence of approximately 23.5% and reported rates ranging from 3.6% to 41.8% depending on membrane thickness, the presence of sinus septa, and the instrumentation used [8]. Other recognized complications include postoperative sinusitis, graft infection, and healing periods of six to 12 months [5,6,9].

Short implants, defined by the European Association for Osseointegration as implants with an intraosseous length of 6 mm or less, offer an alternative that avoids sinus augmentation altogether by allowing direct placement in the residual bone. Advances in surface treatment, thread design, and implant geometry have broadened their clinical indications. Although reduced implant length may theoretically alter occlusal load distribution, short implants simplify surgery, reduce morbidity, shorten treatment duration, and lower overall costs [1,10,11]. Length terminology varies across the literature, and the operational definitions used here, ≤6 mm for short implants and >8 mm for standard implants, leave implants of 6 to 8 mm outside both categories. This gap is deliberate: it isolates the clinical situation in which the two approaches are genuinely alternatives. In practice, the trials eligible for this review compared 5 to 6 mm implants with 10 to 15 mm implants, and no eligible trial tested an intermediate length, so the comparison reported here concerns the two extremes of the range rather than the full continuum of implant lengths.

Nevertheless, the long-term clinical efficacy of short implants remains debated. Earlier studies suggested survival rates inferior to those of standard implants, whereas more recent publications contradict these conclusions. These discrepancies reflect differing methodologies, variable inclusion criteria, and heterogeneous follow-up periods, justifying a rigorous synthesis of the available randomized evidence [2,12,13].

The primary objective of this systematic review and meta-analysis is to compare the clinical efficacy of short implants (≤6 mm) with sinus floor elevation combined with standard implants (>8 mm) in the atrophic posterior maxilla, with implant survival as the principal outcome. The comparison is deliberately restricted to trials in which the control arm involved sinus floor elevation so that the synthesis addresses the specific clinical decision faced when subantral bone height is insufficient. Secondary objectives are to compare marginal bone loss and intraoperative and postoperative complications between the two approaches and to assess the methodological quality of the included trials using RoB 2 (Cochrane, Bristol, UK). The null hypothesis states that implant survival does not differ between the two techniques. The review examines whether the available randomized evidence can distinguish between them on this outcome and whether surgical complexity, morbidity, treatment duration, and cost better guide the therapeutic choice.

## Review

Protocol and registration

This systematic review followed the Preferred Reporting Items for Systematic Reviews and Meta-Analyses (PRISMA) 2020 statement. The protocol was prospectively registered in PROSPERO on 2 April 2026 (registration number CRD420261358691; available at https://www.crd.york.ac.uk/PROSPERO/view/CRD420261358691) before data extraction to ensure transparency and reduce reporting bias.

Search strategy

A systematic search was performed across four electronic databases: MEDLINE (via PubMed), Cochrane CENTRAL, Web of Science, and Scopus. Randomized controlled trials published between 2014 and 2025 were sought that compared short implants placed without sinus floor elevation with standard implants placed with sinus floor elevation in patients with posterior maxillary atrophy. The searches were last run in February 2026, and no update was performed thereafter. Table 1 presents the full search strings and applied filters for each database.

**Table 1:** Literature search strategy

Database	Search terms	Filters
PubMed/MEDLINE	("short dental implants"[MeSH] OR "short implants") AND ("sinus floor augmentation"[MeSH] OR "sinus lift" OR "maxillary sinus elevation") AND ("randomized controlled trial"[pt] OR "prospective")	Humans; English/Spanish; 2014-2025
Cochrane CENTRAL	("short implants") AND ("sinus lift" OR "sinus floor elevation" OR "maxillary sinus augmentation")	Clinical trials; 2014-2025
Web of Science	TS=("short implants" AND ("sinus lift" OR "sinus elevation") AND "atrophic maxilla")	Article; 2014-2025
Scopus	TITLE-ABS-KEY(("short implants") AND ("sinus" OR "sinus lift") AND "posterior maxilla")	RCTs and controlled studies; 2014-2025

Publications from the preceding five years were prioritized for secondary sources such as comparative reviews and meta-analyses. The randomized controlled trials constituting the primary data for this systematic review and meta-analysis were published between 2017 and 2024, as these trials directly compare both techniques in the atrophic posterior maxilla; their inclusion is justified by the absence of more recent alternatives with a comparable study design. For this reason, the 2014-2025 search window was maintained. Reference lists of previously published reviews and of the included studies were additionally hand-searched.

Eligibility criteria

Inclusion and exclusion criteria were defined a priori using the PICOS framework, as detailed in Table 2. Eligible studies enrolled adult patients aged 18 years or older with posterior maxillary atrophy (subantral bone height <10 mm), compared short implants (≤6 mm) placed without sinus floor elevation versus standard implants (>8 mm) placed with sinus floor elevation via either a lateral or transcrestal approach, and reported implant survival, marginal bone loss, or biological and prosthetic complications, with a minimum follow-up of 12 months.

**Table 2:** PICOS eligibility criteria

PICOS component	Description
P - population	Adult patients aged ≥18 years with posterior maxillary atrophy (subantral bone height <10 mm)
I - intervention	Short implants (≤6 mm) placed without sinus floor elevation
C - comparison	Standard implants (>8 mm) placed in conjunction with sinus floor elevation (lateral or transcrestal approach). Trials in which the control arm did not involve sinus floor elevation, including comparisons with longer implants in native bone of adequate height or in bone augmented by other techniques, were excluded
O - outcomes	Implant survival, marginal bone loss, biological and prosthetic complications
S - study design	Randomized controlled trials; follow-up ≥12 months; published 2014-2025

Studies were excluded if they met any of the following criteria: retrospective design; case-control or cross-sectional design; case reports or case series with fewer than 10 participants; use of alternative materials or innovative technologies without a comparable control group; review articles without original data; follow-up shorter than 12 months; or, critically, trials in which the control arm did not involve sinus floor elevation. This last criterion excluded several otherwise high-quality randomized trials comparing short implants with longer implants placed in native bone of adequate height or in bone augmented by techniques other than sinus floor elevation, as these do not address the review question.

One trial initially considered for inclusion (Shi et al., a three-arm randomized trial comparing 6-mm implants alone, 8-mm implants with osteotome sinus floor elevation, and 10-mm implants with osteotome sinus floor elevation) was ultimately excluded because we could not obtain the full text. The event counts, denominators, and handling of participants lost to follow-up could therefore not be verified against the source publication. Because this trial used a three-arm design that required a decision on comparator selection, we judged inclusion based on abstract data alone inappropriate.

Study selection and data extraction

Two authors independently re-extracted all quantitative data in duplicate from the full text of each source publication, tracing each value to its specific table or section. Discrepancies were resolved by returning to the source. When we could not obtain the full text, we extracted no quantitative data and excluded the trial from the pooled analysis.

Data extracted from each eligible publication included author, year of publication, study design, number of patients per treatment group, follow-up duration, implant survival rate, marginal bone loss, and reported complications. Where multiple publications reported outcomes from the same patient cohort at successive follow-up intervals, we used only the most recent available follow-up for the quantitative meta-analysis to prevent double-counting patients.

Risk of bias assessment

The risk of bias of the included trials was assessed using RoB 2 across its five domains: the randomization process, deviations from intended interventions, missing outcome data, measurement of the outcome, and selection of the reported result. Each cohort was assigned an overall judgment according to the RoB 2 algorithm.

Statistical analysis

Quantitative synthesis was restricted to implant survival, the primary outcome. Survival was analyzed at the patient level in the primary analysis, since the included trials differ in the number of implants placed per patient, and patient-level analysis avoids the clustering induced by multiple implants in the same individual; an implant-level analysis is reported as a sensitivity analysis. For each trial, the denominator was the number of randomized patients in each arm, and the numerator was that denominator minus confirmed implant failures; participants lost to follow-up were therefore counted as survivors in every cohort, a conservative and uniform convention applied identically to both arms.

Pooling was performed using a random-effects model, implemented in Python 3.11 (Python Software Foundation, Wilmington, DE, USA) with the NumPy 2.4 (NumPy Project (NumFOCUS, Inc.), Austin, TX, USA) and SciPy 1.17 (SciPy Project (NumFOCUS, Inc.), Austin, TX, USA) libraries. Survival was expressed as risk ratios (RR) with 95% confidence intervals (CIs), computed on the logarithmic scale. Between-study variance was estimated by the DerSimonian-Laird method, as specified in the registered protocol, but CIs are reported using the Hartung-Knapp adjustment. This adjustment was introduced at revision and constitutes a declared deviation from the registered protocol: with only two trials contributing to the primary estimate, the normal-quantile interval underlying the DerSimonian-Laird method is anticonservative, and the Hartung-Knapp interval, based on the t distribution with k − 1 degrees of freedom, is the more honest expression of the uncertainty. The unadjusted DerSimonian-Laird interval is reported alongside it for comparability with the existing literature. Trials with no failures in either arm carry zero weight in an RR analysis and were excluded from the primary pooled estimate; such trials are retained in the qualitative synthesis and reintroduced in a sensitivity analysis using a continuity correction of 0.5. Between-study heterogeneity was quantified using Cochran's Q statistic, τ², and the I² index. Statistical significance was set at p < 0.05. Because survival above 95% in both arms compresses the RR toward unity, results are additionally reported on the failure scale using the Peto odds ratio, the Mantel-Haenszel RR without continuity correction, and the Mantel-Haenszel risk difference, these methods being appropriate to sparse binary events.

Follow-up duration differed across the included cohorts, with outcomes reported at 36 months in two and at 120 months in one. Pooling was performed on the latest follow-up available for each cohort, because the data represent the most complete observation of implant performance in each trial. This rests on the assumption that the relative risk of implant failure is approximately stable over time, an assumption the present data do not support: the two pooled cohorts differ in the direction of effect, and the only cohort favoring standard implants has the longest follow-up. Because only two cohorts contribute to the primary estimate, excluding either leaves a single trial and does not constitute a meta-analysis. Sensitivity analysis therefore focused on the unit of analysis, handling of the event-free cohort, and the choice of variance estimator and interval construction, including the Paule-Mandel estimator and a fixed-effect Mantel-Haenszel model.

Marginal bone loss and complication rates were synthesized narratively rather than pooled. Beyond the lack of consistent measures of dispersion, the included trials measured marginal bone loss using different radiographic methods and reported it with different summary statistics at different time points, so the reported values are not directly comparable across cohorts. Reporting effect estimates for these outcomes without the underlying variance data would convey a false impression of precision, so a narrative synthesis was more appropriate. The extraction sheet, recording every survival count and denominator alongside the source table or section from which it was taken, and the analysis scripts are available from the corresponding author on request.

Generative artificial intelligence (Claude, Anthropic PBC, San Francisco, CA, USA; successive versions including Claude Opus 5, used between January 2026 and June 2026) was used to assist with translation from Spanish, language editing, the computational implementation of the pooling in Python, and, at revision, the transcription of values from the full texts of the included reports into the extraction sheet. No artificial intelligence tool was used to select studies, judge eligibility, assess risk of bias, or draw the conclusions. The computational verification performed at the original submission covered the pooling arithmetic only and did not extend to the fidelity of the extracted data against the source publications; all data have since been re-extracted in duplicate by two authors working independently, directly from the sources, and verified by a human author against the primary publications.

Study selection and baseline characteristics

The electronic search identified 1,247 records across the four databases (PubMed: 485; Cochrane CENTRAL: 218; Web of Science: 304; Scopus: 240), supplemented by 12 additional records from hand-searching, for a total of 1,259 records. After removing 384 duplicates, 875 records were screened by title and abstract, and 803 were excluded. Of the 72 reports sought for retrieval, three could not be obtained, and 69 were assessed in full text; 65 were excluded, most commonly because the control arm did not involve sinus floor elevation or because the publication reported duplicate data from a cohort already considered. Four publications corresponding to three independent patient cohorts were finally included (Figure 1) [10,14-16]. A fifth report of one of these cohorts could not be retrieved and contributes no data [9].

**Figure 1:**
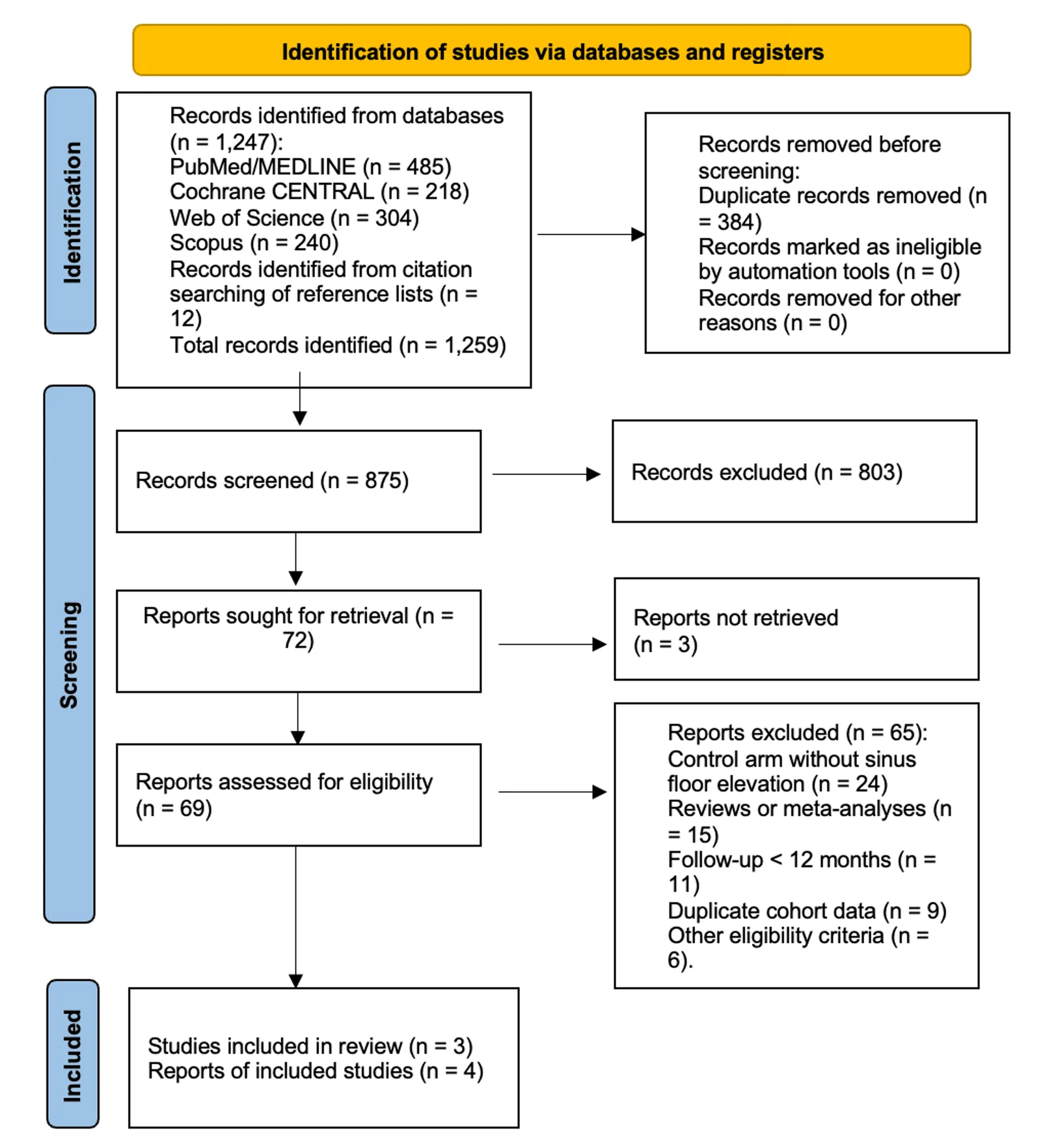
PRISMA 2020 flow diagram of the study selection process Records identified through citation searching of reference lists (n = 12) were combined with database records at the identification stage; we then deduplicated the merged set and applied all subsequent steps. Reports whose full text could not be obtained (n = 3) were recorded as not retrieved and were not assessed for eligibility; these comprise the two reports of the Shi trial and the three-year report of cohort A [9]. PRISMA: Preferred Reporting Items for Systematic Reviews and Meta-Analyses

The three independent cohorts comprised 174 randomized patients. Follow-up was 36 months in two cohorts and 120 months in one (Table 3). One cohort (Cohort A) recorded no implant failures in either arm and is described qualitatively but does not enter the primary pooled estimate; therefore, the primary estimate rests on 154 patients (83 short implants/71 standard implants with sinus floor elevation). Risk of bias assessment using RoB 2 is summarized in Table 4 and Figure 2.

**Table 3:** Reports of the three included cohorts (four publications) The review includes three cohorts reported in the four publications listed here; cohort C appears in two publications, with the more recent one providing the data used in the synthesis. A further report of cohort A (the three-year report [9]) could not be retrieved; it is recorded as not retrieved in Figure 1 and contributes no data. * Data point used for the quantitative meta-analysis. Cohorts are labeled A, B, and C to match the forest plot. ShI = short implants (≤6 mm); SI = standard implants (>8 mm) with sinus floor elevation; MBL = marginal bone loss. Survival is reported at the patient level throughout, with denominators corresponding to randomized patients per arm; participants lost to follow-up are counted as survivors. Cohort A randomized 20 patients in total (10 per arm). MBL is reported as each source publication reports it, and the values are not comparable across cohorts: ᵃ patient level, mean ± SD, periapical radiographs (paralleling technique); ᵇ implant level, mean (95% CI), panoramic radiographs; ᶜ implant level, mean ± SD, periapical radiographs (paralleling technique); ᵈ patient level, median (min; max), periapical radiographs (Rinn holders). p-values are those reported in each source and derive from different tests. Cohort A recorded no implant failures in either arm at the follow-up used and therefore carries no weight in an RR analysis. The two failures in cohort B occurred in a single control-arm patient at two months, before prosthetic loading, following chronic sinus infection with complete graft loss. The two failures in cohort C occurred in the short-implant arm, between four and seven years, for undetermined reasons. CI: confidence interval, SD: standard deviation, RR: risk ratio

Cohort	Author	Year	n ShI	n SI	Follow-up	Survival ShI	Survival SI	MBL ShI (mm)	MBL SI (mm)	p (MBL)
A	Felice et al., 2015 [10]^*^	2015	10	10	12 months	100% (10/10)	100% (10/10)	0.70 ± 0.19^a^	0.87 ± 0.21^a^	0.078
B	Bechara et al., 2017 [14]^*^	2017	33	20	36 months	100% (33/33)	95.0% (19/20)	0.20 (0.17–0.24)^b^	0.27 (0.23–0.31)^b^	0.01
C	Pohl et al., 2017 [15]	2017	50	51	36 months	100% (50/50)	100% (51/51)	0.44 ± 0.44^c^	0.45 ± 0.55^c^	0.82
C	Thoma et al., 2024 [16]​​​​​​​^*^	2024	50	51	120 months	96.0% (48/50)	100% (51/51)	0.00 (0.00; 3.25)^d^	0.00 (0.00; 4.55)^d^	0.73

**Table 4:** Risk of bias assessment (RoB 2) Risk of bias is assessed per cohort (n = 3), not per publication, since the multiple reports of a single cohort describe one randomized trial. D1-D5 are the five RoB 2 domains. Cohort A was assessed on Felice et al. [10], the one-year report of that trial and the data point used in the quantitative synthesis; the full text of Gastaldi et al. [9], the three-year report of the same trial, could not be retrieved and was therefore not assessed. No prospective trial registration is reported for cohort A, affecting the reported-result domain. In cohort B, randomization of 53 participants produced arms of 33 and 20, and no prospective trial registration or published protocol is reported, affecting the randomization and reported-result domains. Cohort C was prospectively registered (NCT01030523) and used block randomization, but 21% of participants were lost between the 5- and 10-year follow-ups, affecting the missing-outcome-data domain. Blinding of outcome assessment was not possible in any trial because implant length differed between arms; this was not judged to compromise the measurement domain, as implant loss is an objective outcome.

Cohort	D1: randomization	D2: deviations	D3: missing data	D4: measurement	D5: reported result	Overall
Felice et al., 2015 [10] (A)	Low	Low	Low	Low	Some concerns	Some concerns
Bechara et al., 2017 [14] (B)	Some concerns	Low	Low	Low	Some concerns	Some concerns
Pohl et al., 2017 [15] (C)/Thoma et al., 2024 [16] (C)	Low	Low	Some concerns	Low	Low	Some concerns

**Figure 2:**
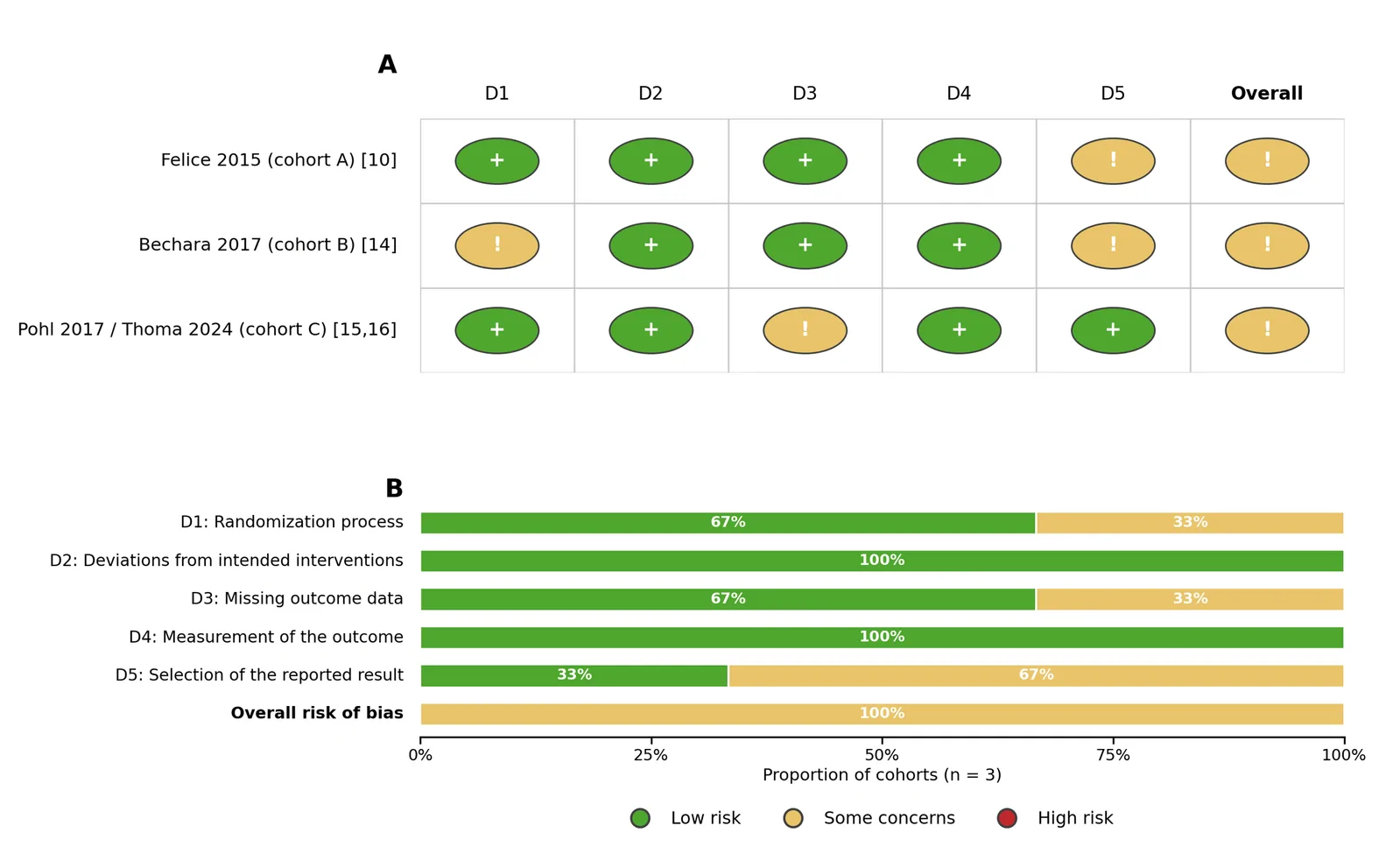
Risk of bias in the included cohorts (RoB 2) (A) Traffic-light plot by domain and cohort. (B) Weighted summary across the three included cohorts. D1, randomization process; D2, deviations from intended interventions; D3, missing outcome data; D4, outcome measurement; D5, selection of the reported result. Assessment is per cohort (n = 3), not per publication. The judgments shown are those of Table 4, where the reasoning for each is given.

Implant survival

Cumulative patient-level survival across the three cohorts was 97.8% (91/93) for short implants and 98.8% (80/81) for standard implants with sinus floor elevation. Three implant failures occurred across 174 randomized patients.

Meta-analysis of the two event-contributing cohorts yielded a pooled RR of 1.00 (95% CI: 0.56-1.76) under the Hartung-Knapp adjustment, indicating that the available data cannot discriminate between the two approaches (Figure 3). The unadjusted DerSimonian-Laird interval was 0.91-1.09 (p = 0.92); the difference in width between the two, at an unchanged point estimate, follows from the fact that with two trials the relevant quantile is t₁ = 12.71 rather than 1.96, and the adjusted interval is reported as primary throughout. Between-study heterogeneity was substantial (Q = 2.45, df = 1, p = 0.12; I² = 59%; τ² = 0.0025).

**Figure 3:**
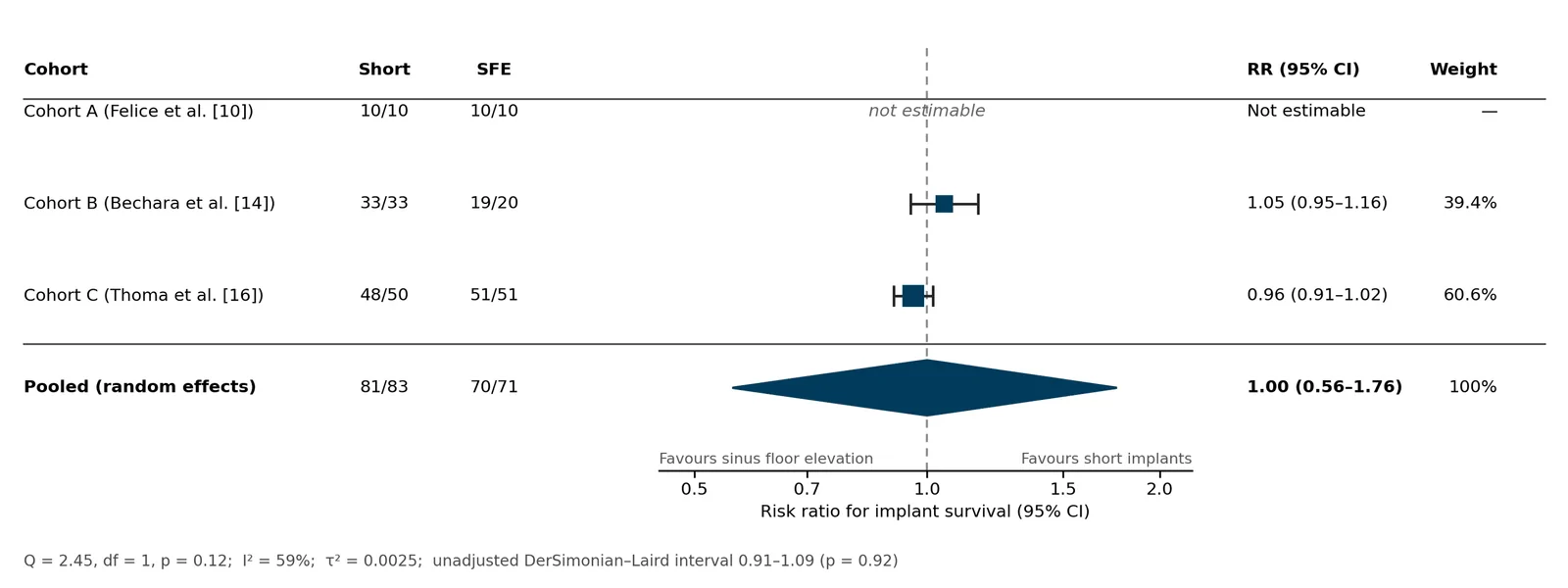
Forest plot of implant survival showing short implants and SFE Random-effects model, DerSimonian-Laird variance estimator with Hartung-Knapp adjustment: pooled RR 1.00 (95% CI: 0.56-1.76); unadjusted interval 0.91-1.09 (p = 0.92); Q = 2.45, df = 1, p = 0.12; I² = 59%; τ² = 0.0025. The plot displays the three included cohorts, not the four publications reporting them. Survival is analyzed at the patient level, with denominators corresponding to randomized patients per arm. Squares represent individual cohort RR, sized by inverse-variance weight; horizontal lines represent 95% CI; the diamond represents the pooled estimate. The dashed vertical line marks the null value (RR = 1.00). Cohort A (Felice et al. [10]) appears without a point estimate or CI because none exists: no implant failure occurred in either arm (10/10 versus 10/10), so the RR has zero variance on the logarithmic scale, carries no information about a relative risk, and cannot be weighted under any inverse-variance model. It is displayed rather than omitted so that the figure accounts for all three included cohorts. A continuity correction of 0.5 would allow it to be plotted, but would assign it a precision it does not have; that option is reported as a sensitivity analysis instead and yields a pooled RR of 0.99 (95% CI: 0.93-1.05). SFE: sinus floor elevation, CI: confidence interval, RR: risk ratio

The two cohorts point in opposite directions. Bechara et al. [14] reported 100% survival with short implants versus 95.0% with sinus floor elevation (RR = 1.05; 95% CI: 0.95-1.16), with the two failures occurring in a single control-arm patient at two months, before prosthetic loading, following chronic sinus infection with complete graft loss. Thoma et al. [16] reported 96.0% versus 100% at 10 years (RR = 0.96; 95% CI: 0.91-1.02), with both failures occurring in the short-implant arm for undetermined reasons between four and seven years. With three implant failures in total, this divergence cannot be distinguished from random variation, and neither trial individually reaches statistical significance.

Four sensitivity analyses were performed. Reintroducing the event-free cohort with a continuity correction of 0.5 yielded a pooled RR of 0.99 (95% CI: 0.93-1.05) under the unadjusted model and 0.99 (0.87-1.12) under the Hartung-Knapp adjustment, with I² = 20%. Repeating the primary analysis at the implant level yielded 1.00 (95% CI: 0.93-1.08; p = 0.92; I² = 74%). Substituting the Paule-Mandel estimator returned the same between-study variance as DerSimonian-Laird (τ² = 0.0025) and therefore the same interval, as expected at this number of trials and indicating that interval width is driven by interval construction rather than the variance estimator. A fixed-effect Mantel-Haenszel model yielded 0.99 (0.94-1.04) but is not appropriate given the observed heterogeneity. No analysis materially alters the point estimate, and none supports a claim of precision.

Marginal bone loss

Marginal bone loss consistently favored short implants across all verifiable comparisons, but the magnitude was small, and the difference reached statistical significance in only one cohort. Bechara et al. [14] reported 0.20 mm versus 0.27 mm at three years (p = 0.01). Felice et al. [10] reported 0.70 mm versus 0.87 mm at one year, a 0.17 mm difference that did not reach significance (p = 0.078). Pohl et al. [15] found essentially identical values at three years (0.44 mm versus 0.45 mm; p = 0.82), and Thoma et al. [16] found a median bone level of 0.00 mm in both groups at ten years (p = 0.73), with no significant influence of implant length, crown height space, or crown-to-implant ratio on bone level change.

These values are not directly comparable across trials. Bechara et al. measured bone levels on panoramic radiographs, a method the authors themselves identify as subject to distortion, whereas the other trials used periapical radiographs with a paralleling technique. The trials also report different summary statistics, at different time points, and using different units of analysis. Therefore, a pooled mean difference was not calculated, and no formal test of effect across trials is available.

Taken together, the data provide no evidence that short implants are associated with greater marginal bone loss than standard implants placed after sinus floor elevation. This consistency rests on the direction of point estimates rather than a formal test, and in the two cohorts with the longest follow-up, the between-group difference was negligible.

Complications

Biological Complications

Biological complications were consistently less frequent in the short-implant group. Bechara et al. [14] reported 19 biological complications in the control arm versus none in the test arm: three episodes of intraoperative bleeding, fifteen of postoperative swelling, and one chronic sinus infection with complete graft loss, which caused the only two implant failures recorded in a standard-implant arm across the entire review. Thoma et al. [16] reported peri-implantitis in 4.2% of short-implant patients versus 13.3% of patients in the standard-implant group at ten years, a difference that did not reach statistical significance (p = 0.37). The complications concerned are structurally specific to sinus floor elevation: perforation of the Schneiderian membrane, postoperative sinusitis, and graft infection, and are absent by construction from the short-implant approach.

Prosthetic and Technical Complications

Bechara et al. [14] reported no prosthetic complications in either group over three years. Thoma et al. [16] reported technical complications affecting 38.0% of patients in the short-implant group and 45.1% in the standard-implant group over ten years; screw loosening was most frequent, observed in 20 short and 13 standard implants. The mean crown-to-implant ratio in the short-implant group of that trial was 1.86 ± 0.23, and the authors found no statistically significant influence of the ratio on bone level change; the present data do not, however, support a specific threshold below which prosthetic risk is unaffected.

As with marginal bone loss, these findings are reported narratively. Definitions of biological complications varied across trials, and denominators were reported inconsistently at the patient and implant level, precluding valid quantitative pooling.

Discussion

This systematic review and meta-analysis found no statistically significant difference in implant survival between short implants and standard implants combined with sinus floor elevation (RR = 1.00; 95% CI: 0.56-1.76). This absence of a detected difference should not be read as demonstrated equivalence: three implant failures across 174 randomized patients provide very little power, and the two pooled cohorts diverge in the direction of effect (I² = 59%). On the failure scale, the same data give a Peto odds ratio of 1.70 (95% CI: 0.17-16.86), an interval compatible with a failure risk ranging from approximately one fifth to 17 times that of the comparator. The near-unity RR on the survival scale should not be read as indicating near-identity of the two approaches. The point estimate is consistent with previous meta-analyses, including those of Fan et al. [12], Starch-Jensen et al. [2], Yan et al. [13], and Ravidà et al. [17]. Still, the present synthesis is smaller and more narrowly defined than any of them, and its CI is considerably wider.

Short implants have a more favorable morbidity profile by eliminating the complications inherent to sinus floor elevation. This potential advantage may be particularly relevant in older patients, patients receiving multiple medications, or immunocompromised patients. The Cochrane review by Esposito et al. [6] noted this trend, which we confirm here with updated data.

Regarding marginal bone loss, the evidence is weaker than a uniform effect would suggest: one cohort found a statistically significant but clinically negligible advantage for short implants, whereas the other cohorts found no significant difference. Survival showed a divergent long-term signal: the 10-year data from Thoma et al. [16] report 96.0% survival with short implants versus 100% with sinus grafting, whereas the three-year data from Bechara et al. [14] report the reverse pattern. The available evidence cannot determine whether this reflects a genuine time-dependent effect or the instability inherent in the small number of events in each trial, and it is the clearest argument for randomized trials with extended follow-up.

Prosthetic complications did not differ detectably between groups, providing no support for the hypothesis of increased risk associated with an unfavorable crown-to-implant ratio. Compared with the 2015 EAO consensus statement [11], this review provides updated data through 2024, including the only trial with 10-year follow-up [16].

Strengths and Limitations

This review follows PRISMA and RoB 2, uses independent duplicate screening and re-extraction of all quantitative data against the source publications, reports several sensitivity analyses, and includes only randomized controlled trials. Two features strengthen the synthesis's internal validity: the exclusive use of the latest follow-up per cohort, which avoids double-counting patients, and the strict requirement that the control arm involve sinus floor elevation, which excluded trials comparing short implants with longer implants in native or otherwise augmented bone. This deliberately conservative approach reduced the sample but ensured that every included comparison directly addresses the review question.

These restrictions lead to the principal limitations. Only two independent cohorts, totaling 154 patients, contributed to the pooled estimate, with three implant failures in total. This severely limits power, precludes formal assessment of publication bias, and makes the pooled estimate sensitive to any single event. Substantial heterogeneity was present (I² = 59%), with the two cohorts showing effects in opposite directions; with two trials, this heterogeneity cannot be explored by subgroup or meta-regression. With this number of trials, between-study variance cannot be estimated with useful precision, and CIs based on normal quantiles are anticonservative; therefore, the primary interval is reported with the Hartung-Knapp adjustment, and its width (0.56 to 1.76) is compatible with a substantial advantage to either approach. The narrower interval obtained under the unadjusted model should not be relied upon. A third cohort recorded no failures in either arm and therefore carries no information about an RR. The three-year report for that cohort could not be retrieved, as the journal ceased publication in 2018; the cohort is represented by its one-year report, which meets the review's minimum follow-up criterion and was verified in full. Since no failures occurred in either arm at either time point, this does not affect the pooled estimate. Still, it shortens the observation window described for that cohort and constitutes a deviation from the registered protocol, which specified the most recent follow-up available. The 10-year data rest on a single trial. Participants lost to follow-up were counted as survivors in every cohort, which inflates survival in both arms, most notably in the trial with 21% attrition between five and ten years. Definitions of a "short" implant, radiographic methods for measuring bone level, and definitions of biological complications varied across authors. Finally, one relevant three-arm randomized trial could not be included because its full text could not be retrieved; its published abstract reports lower survival for 6-mm implants than for 10-mm implants with sinus floor elevation, and its inclusion might therefore have shifted the pooled estimate.

Certainty of the Evidence

Applying the GRADE approach, certainty for implant survival is very low. The evidence derives from randomized trials and therefore starts at high certainty but is downgraded for risk of bias, as both contributing trials were rated ‘some concerns’ overall; inconsistency, as the two trials point in opposite directions with I² = 59%; and serious imprecision, as three events in total yield an interval compatible with a substantial advantage to either approach. Certainty for marginal bone loss and prosthetic complications is likewise very low, with no pooled estimate available and measurement methods differing across trials. Certainty is low rather than very low for biological complications, where the direction is consistent across trials, and the complications concerned are structurally specific to sinus floor elevation. Publication bias could not be formally assessed for any outcome.

Clinical Recommendations

The considerations that follow do not derive from the present synthesis, which did not stratify outcomes by residual bone height or by crown-to-implant ratio and could not have done so with three cohorts. They are drawn from the eligibility criteria of the included trials and from external sources and are offered as contextual guidance rather than as findings of this review. The included trials enrolled patients with residual subantral bone height of 5 to 6 mm in two cohorts and 5 to 7 mm in the third, which is the range over which the comparison reported here was actually tested; below that range, primary stability for a 6-mm implant becomes the limiting factor and sinus floor elevation remains the established option. Short implants are of particular interest in elderly or polymedicated patients, including those receiving anticoagulant therapy or antiresorptive agents, in immunocompromised patients, and in those for whom a prolonged or additional surgical procedure is undesirable, as well as for patients declining invasive surgery or seeking shorter treatment timelines. This review found biological complications markedly more frequent after sinus floor elevation, and that finding bears directly on the choice. Regarding the crown-to-implant ratio, the mean value in the short-implant arm of the trial with 10-year follow-up was 1.86, and the authors of that trial found no significant influence of the ratio on bone level change [16]; the present data do not identify a threshold, and the frequently cited 2:1 limit should be understood as a convention rather than a value established by this synthesis. The choice of approach should be individualized according to the patient's systemic status, residual anatomy, prosthetic expectations, and the cost-benefit balance.

Future Perspectives

The small number of eligible trials identified by this review is itself a finding. Despite the clinical importance of the question, only three independent randomized cohorts have directly compared short implants with sinus floor elevation in the atrophic posterior maxilla, and only two provide information on relative failure risk. Further randomized controlled trials with follow-up beyond 10 years and standardized biological, clinical, and economic endpoints are needed. International implant registries would facilitate long-term surveillance of both techniques. Future studies should include cost-effectiveness analyses, evaluate emerging technologies such as computer-assisted surgical navigation and CBCT-based digital planning, and assess the clinical performance of ultrashort implants (<5 mm), for which evidence remains limited.

## Conclusions

This systematic review of three independent randomized cohorts totaling 174 patients, with two cohorts totaling 154 patients contributing to the meta-analysis, found no statistically significant difference in implant survival between short implants and standard implants placed with sinus floor elevation (RR = 1.00, 95% CI: 0.56-1.76). With three implant failures in total and substantial heterogeneity between the two contributing cohorts, the CI is compatible with a substantial advantage to either approach, and the review cannot determine whether the two techniques differ in implant survival. Marginal bone loss favored short implants in one cohort by a clinically negligible margin and did not differ significantly in the other cohorts. The most consistent finding of this review concerns morbidity rather than survival. Every failure recorded in a standard-implant arm was attributable to a graft-related complication, and biological complications were markedly more frequent after sinus floor elevation. Based on the current evidence, short implants are a reasonable alternative in the atrophic posterior maxilla, and the therapeutic choice should therefore be guided by surgical burden, morbidity, treatment duration, and cost, given that the available randomized evidence cannot distinguish between the two techniques in terms of survival.
